# HIV‐1 Vpr Drives a B Cell‐Like Phenotype in Primary CD4^+^ T Cells

**DOI:** 10.1002/mco2.71018

**Published:** 2026-09-27

**Authors:** Peipei Wang, Jieying Li, Wei Wang, Zhuoyue Meng, Huixin Wu, Shumin Du, Kai Deng, Jinfeng Cai

**Affiliations:** ^1^ Department of Infectious Diseases The Third Affiliated Hospital Sun Yat‐sen University Guangzhou China; ^2^ Basic Medical Experimental Teaching Center Zhongshan School of Medicine Sun Yat‐sen University Guangzhou China; ^3^ Institute of Human Virology Zhongshan School of Medicine Key Laboratory of Tropical Disease Control of Ministry of Education Sun Yat‐sen University Guangzhou China; ^4^ Department of Immunology and Microbiology Zhongshan School of Medicine Sun Yat‐sen University Guangzhou China; ^5^ Core Facility of Medical Science Sun Yat‐sen University Guangzhou China; ^6^ Advanced Medical Technology Center Zhongshan School of Medicine The First Affiliated Hospital Sun Yat‐sen University Guangzhou China

**Keywords:** B cell‐like phenotype, CD4^+^ T cells, HIV‐1, transcriptional reprogramming, Vpr

## Abstract

CD4^+^ T cells are the principal cellular targets of HIV‐1, yet the extent to which viral proteins reshape their transcriptional identity remains incompletely understood. Here, using an integrated multi‐omics approach, we identified a distinct population of HIV‐1 RNA‐positive CD4^+^ T cells that localized within transcriptionally defined B‐cell clusters and expressed multiple canonical B cell‐associated genes during acute infection. Mechanistic analyses revealed that this phenotype was driven by the HIV‐1 accessory protein Vpr. Both Vpr overexpression and Vpr‐deficient viral systems demonstrated that Vpr induces the expression of CD19, MS4A1, CD22, and PAX5 in primary CD4^+^ T cells. Single‐cell RNA sequencing, bulk RNA sequencing, and assay for transposase‐accessible chromatin using sequencing (ATAC‐seq) further showed that Vpr promotes extensive transcriptional and epigenetic remodeling, accompanied by increased accessibility and expression of genes involved in B‐cell differentiation and development, including PAX5, EBF1, and PRDM1. Notably, this B cell‐like transcriptional program was transient and gradually diminished during prolonged culture. Together, our findings uncover an unexpected role of Vpr in inducing lineage‐associated transcriptional reprogramming in mature CD4^+^ T cells and provide new insight into how HIV‐1 manipulates host‐cell identity during infection.

## Graphical abstract

During acute HIV‐1 infection, Vpr induces transcriptional and epigenetic remodeling of primary CD4^+^ T cells, activating a transient B cell‐like program characterized by CD19, MS4A1, CD22, and PAX5 expression and increased chromatin accessibility at lymphocyte differentiation genes. This remodeling promotes the emergence of HIV‐1 RNA^+^ B‐like cells, revealing a previously unrecognized mechanism by which HIV‐1 reshapes host‐cell identity. The figure was created with elements from MedPeer (www.medpeer.cn).

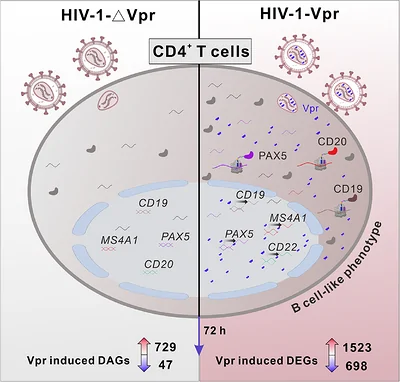

## 1 Introduction

Despite the remarkable success of antiretroviral therapy (ART) in suppressing viral replication, HIV‐1 infection remains incurable due to the persistence of long‐lived latent reservoirs that can reinitiate viremia upon treatment interruption [1, 2]. These reservoirs are predominantly maintained within CD4^+^ T cell populations, which serve as the primary targets for viral entry, integration, and productive replication [3, 4, 5, 6, 7]. Beyond direct cytopathic effects, HIV‐1 infection accelerates immune cell exhaustion [8, 9], disrupts cytokine production [10, 11], impairs B cell and cytotoxic T lymphocyte (CTL) functions [12, 13, 14, 15], alters cell proliferation and differentiation [16, 17, 18], and compromises immune system renewal and senescence [19, 20, 21]. Importantly, these pathological features cannot be fully explained by viral replication alone, suggesting that individual viral proteins actively reshape host‐cell states and immune function.

Among the most striking yet incompletely understood consequences of HIV‐1 infection is the disruption of B cell homeostasis. Although B cells are not productively infected, individuals living with HIV‐1 exhibit persistent humoral immune abnormalities, including hypergammaglobulinemia, polyclonal B cell activation, expansion of exhausted or atypical memory B cell subsets, and impaired vaccine responsiveness [12, 13]. These observations indicate that HIV‐1 infection exerts broad effects on B cell biology indirectly through infected or bystander immune cells. However, the cellular and molecular mechanisms linking HIV‐1 infection of CD4^+^ T cells to aberrant B cell states remain poorly defined, highlighting a gap in our understanding of virus‐induced immune remodeling.

HIV‐1 encodes a limited number of regulatory and accessory proteins, including Tat, Rev, Vif, Vpr, Vpu, and Nef, which collectively reprogram host cells to facilitate viral replication and immune evasion. While many of these proteins function by antagonizing specific host restriction factors or immune defenses, accumulating evidence suggests that they also broadly alter host transcriptional and signaling networks [22, 23, 24, 25, 26, 27, 28]. In particular, Vpr is unique in being incorporated into virions and delivered into newly infected cells at the earliest stages of infection. Although Vpr is not strictly required for viral replication in vitro, it significantly enhances viral fitness in vivo and has been implicated in diverse cellular processes, including cell cycle arrest, apoptosis regulation, and modulation of host gene expression [29, 30, 31, 32, 33, 34, 35, 36, 37, 38, 39]. Recent studies further suggest that Vpr can induce widespread transcriptional remodeling and alter cellular differentiation states in primary CD4^+^ T cells [40, 41, 42, 43]. However, whether Vpr can drive lineage‐associated transcriptional programs and reshape immune cell identity in a broader context remains unclear.

Understanding how viral proteins such as Vpr reshape host‐cell identity requires approaches capable of resolving heterogeneous and dynamic cellular responses at single‐cell resolution. Bulk assays obscure rare or transient infection‐associated states due to averaging across infected, bystander, and uninfected cells. In contrast, single‐cell multi‐omics technologies enable simultaneous profiling of viral transcripts, host gene expression, and chromatin accessibility at the level of individual cells, providing an opportunity to define virus‐induced cell states with high resolution. Such approaches are particularly well suited to interrogating whether HIV‐1 infection induces previously unrecognized transcriptional programs associated with alternative immune lineages.

In this study, we applied integrated single‐cell multi‐omics to primary CD4^+^ T cells during HIV‐1 infection and identified a distinct population of HIV‐1 RNA‐positive cells that clustered within a transcriptionally defined B cell‐like compartment. We demonstrate that this phenotype is driven by the viral accessory protein Vpr, which induces the expression of canonical B cell‐associated genes, including *CD19, MS4A1 (CD20), CD22*, and *PAX5*. Multi‐layered analyses using single‐cell RNA sequencing, bulk RNA sequencing, and assay for transposase‐accessible chromatin using sequencing (ATAC‐seq) further revealed that Vpr promotes extensive transcriptional and epigenetic remodeling, preferentially activating regulatory networks associated with B cell differentiation and development. Notably, this B cell‐like transcriptional program is transient and diminishes over time, indicating a reversible state of lineage‐associated transcriptional plasticity. Together, these findings uncover an unexpected role for Vpr in reshaping host‐cell transcriptional identity and provide new insight into HIV‐1‐induced immune cell state remodeling.

## 2 Results

### 2.1 Emergence of HIV‐1 RNA^+^ Cells With a B Cell‐Like Transcriptional Signature During Acute HIV‐1 Infection

**FIGURE 1:**
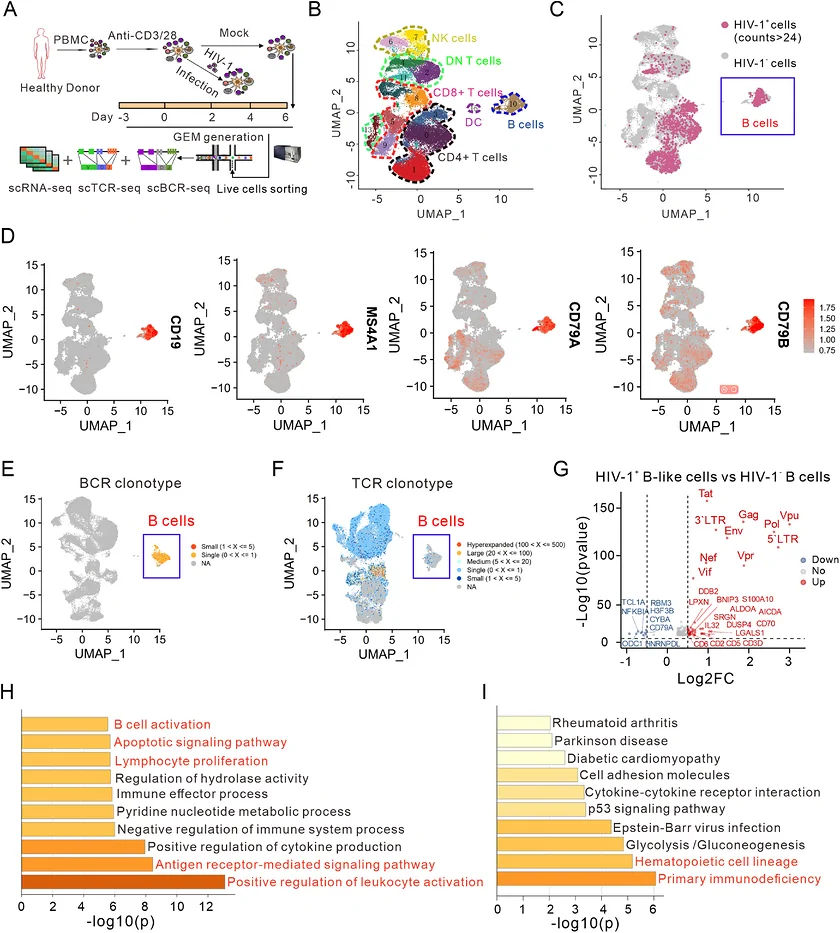
Single‐cell multi‐omics identifies a population of HIV‐1 RNA^+^ B‐like cells during acute HIV‐1 infection. (A) Schematic overview of the experimental workflow. PBMCs isolated from healthy donors were activated, infected with HIV‐1, and subjected to scRNA‐seq, scTCR‐seq, and scBCR‐seq. Uninfected control samples included Day−3 (D−3_NC), Day 0 (D0_NC), and Day 6 (D6_NC). HIV‐1‐infected samples were collected at Day 2 (D2_HIV), Day 4 (D4_HIV), and Day 6 (D6_HIV) post‐infection. (B) UMAP visualization depicting the unsupervised clustering of single‐cell transcriptomic phenotypes from all groups. Each dot represents an individual cell, with distinct colors indicating major cell types. (C) UMAP plot highlighting HIV‐1 RNA^+^ cells identified by detection of HIV‐1 transcripts within the single‐cell datasets. Red dots indicate HIV‐1 RNA^+^ cells. (D) UMAP feature plots showing expression of canonical B‐cell marker genes *CD19, MS4A1 (CD20), CD79A*, and *CD79B*. Gray indicates low or undetectable expression and red indicates high expression. (E) Distribution plot showing the frequencies of different BCR clonotypes across cell clusters. (F) Distribution plot displaying the frequencies of different TCR clonotypes across cell clusters. (G) Volcano plot showing differentially expressed genes (DEGs) between HIV‐1 RNA^+^ B‐like cells and HIV‐1 RNA^−^ cells within the B‐cell cluster. Red dots indicate significantly upregulated genes and blue dots indicate significantly downregulated genes. The x‐axis represents log2 fold change and the y‐axis represents −log10 adjusted p value. (H and I) Functional enrichment analysis of genes upregulated in HIV‐1 RNA^+^ B‐like cells. Bar plots show the top enriched GO biological processes (H) and KEGG pathways (I). Representative pathways related to lymphocyte differentiation and immune responses are highlighted in red.

To investigate how HIV‐1 infection reshapes host‐cell transcriptional programs, we performed single‐cell RNA sequencing (scRNA‐seq), single‐cell T‐cell receptor sequencing (scTCR‐seq), and single‐cell B‐cell receptor sequencing (scBCR‐seq) on peripheral blood mononuclear cells (PBMCs) from healthy donors before and after HIV‐1 infection (Figure 1A). Freshly isolated PBMCs were activated with anti‐CD3/CD28 antibodies for 3 days and subsequently infected with HIV‐1‐ΔE‐GFP pseudotyped with a CXCR4‐tropic HIV‐1 envelope (Figure S1A). Infection efficiency was monitored by flow cytometry (Figure S1B), and live cells were collected at the indicated time points for single‐cell analyses (Figure 1A, Figure S1C). Unsupervised clustering identified 16 immune‐cell populations based on canonical marker genes, including CD4^+^ T cells, CD8^+^ T cells, double‐negative T cells, natural killer cells, B cells, and other immune subsets (Figure 1B, Figure S1D). To identify infected cells, HIV‐1 transcript reads were integrated with the single‐cell transcriptomic datasets. As expected, HIV‐1 RNA^+^ cells were predominantly detected within CD4^+^ T‐cell and double‐negative T‐cell clusters. Unexpectedly, a subset of HIV‐1 RNA^+^ cells was also identified within a cluster annotated by canonical B‐cell markers (Figure 1C, Figure S1E).

To exclude the possibility that this observation resulted from batch‐specific effects, HIV‐1‐infected samples from individual time points were analyzed independently. HIV‐1 RNA^+^ cells with B cell‐like transcriptional features were consistently detected at 2, 4, and 6 days post‐infection, although their relative abundance among total HIV‐1 RNA^+^ cells gradually declined over time (Figure S1F,G). Canonical B‐cell markers, including CD19, MS4A1 (CD20), CD79A, and CD79B, were highly enriched within the B‐like cluster (Figure 1D). Interestingly, a subset of cells within this cluster simultaneously expressed T‐cell‐associated transcripts, including CD3D, CD3E, and CD4 (Figure S1H), suggesting the presence of a hybrid transcriptional state.

To further characterize these HIV‐1 RNA^+^ B‐like cells, we examined antigen receptor clonotypes. As expected, B‐cell receptor (BCR) clonotypes were highly enriched within the B‐like cluster (Figure 1E). Notably, a subset of cells within this cluster also retained detectable T‐cell receptor (TCR) clonotypes (Figure 1F), consistent with the possibility that some cells acquired B‐cell‐associated transcriptional features while retaining characteristics of T cells. Differential gene expression analysis revealed robust expression of HIV‐1 transcripts, including *LTR, Gag, Pol, Env, Tat*, and accessory genes, within HIV‐1 RNA^+^ B‐like cells (Figure 1G). Functional enrichment analyses using Gene Ontology (GO) and Kyoto Encyclopedia of Genes and Genomes (KEGG) pathways demonstrated that genes preferentially upregulated in HIV‐1 RNA^+^ B‐like cells were associated with lymphocyte activation, proliferation, differentiation, and primary immunodeficiency pathways (Figure 1H,I). Collectively, these findings identify a previously unrecognized population of HIV‐1 RNA^+^ cells exhibiting a B cell‐like transcriptional signature during acute HIV‐1 infection and suggest that HIV‐1 infection is associated with substantial remodeling of host‐cell identity programs.

### 2.2 HIV‐1 Vpr Triggers the Expression of B Cell‐associated Genes in Primary CD4^+^ T Cells

**FIGURE 2:**
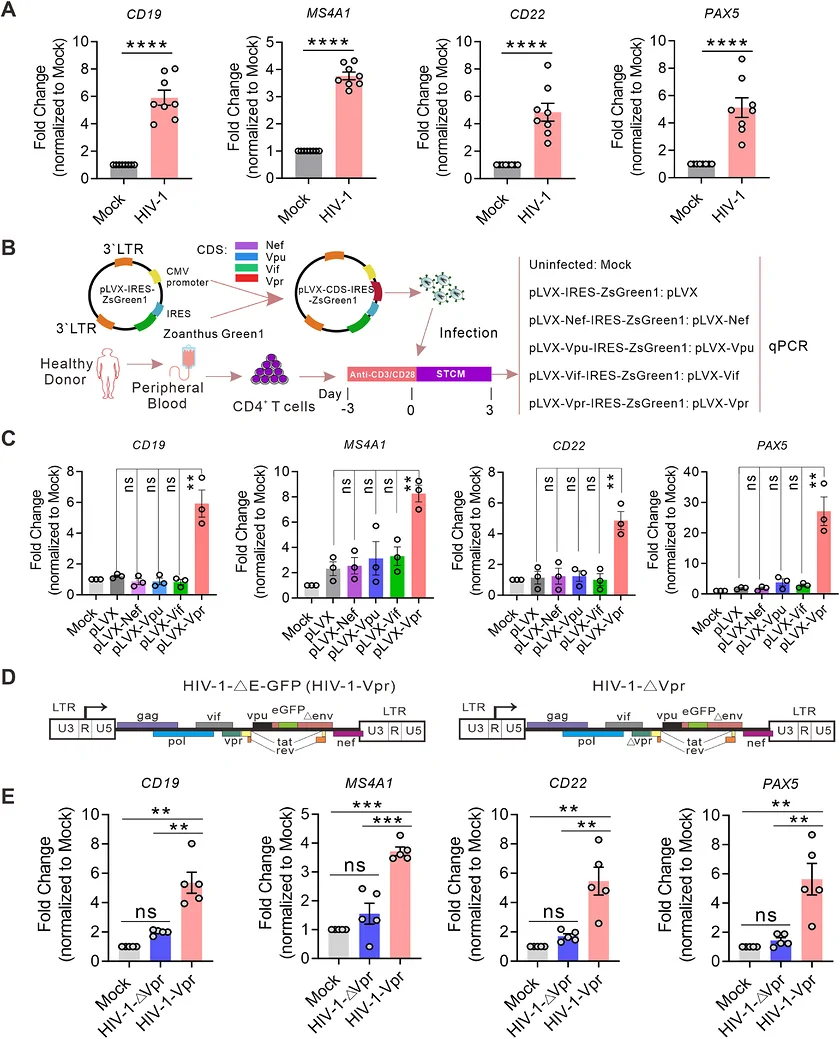
HIV‐1 Vpr specifically induces expression of B cell‐associated genes in primary CD4^+^ T cells. (A) Relative mRNA expression of the B cell‐associated genes *CD19, MS4A1, CD22*, and *PAX5* in primary CD4^+^ T cells infected with HIV‐1 or mock‐treated controls, determined by quantitative PCR (qPCR) (*n* = 8 donors). (B) Experimental scheme for lentiviral overexpression of individual HIV‐1 accessory proteins in activated primary CD4^+^ T cells. (C) qPCR analysis of CD19, MS4A1, CD22, and PAX5 expression in primary CD4^+^ T cells transduced with pLVX‐empty vector, pLVX‐Vpr, pLVX‐Vif, pLVX‐Nef, or pLVX‐Vpu (n = 3 donors). *p* values compare each condition with its pLVX control. (D) Schematic illustration of the generation of HIV‐1‐Vpr and HIV‐1‐ΔVpr viruses derived from the HIV‐1 NL4‐3 molecular clone. (E) Relative expression of *CD19, MS4A1, CD22*, and *PAX5* in activated primary CD4^+^ T cells infected with HIV‐1‐Vpr or HIV‐1‐ΔVpr. CD4^+^ T cells were activated with anti‐CD3/CD28 antibodies for 3 days prior to infection, and total cellular RNA was collected 3 days post‐infection for qPCR analysis (*n* = 5 donors). Data are presented as mean ± s.d. Statistical significance was determined using an unpaired two‐tailed Student's *t* test. ns, not significant; **p < 0.01; ***p < 0.001; ****p < 0.0001.

To investigate the mechanism underlying the emergence of HIV‐1 RNA^+^ cells with B cell‐like transcriptional features, we first sought to validate the induction of B cell‐associated genes following HIV‐1 infection. Consistent with the single‐cell data, infection of primary CD4^+^ T cells resulted in increased expression of canonical B cell markers, including *CD19, MS4A1, CD22*, and *PAX5* (Figure 2A). To determine whether specific HIV‐1 accessory proteins contributed to this phenotype, we generated lentiviral expression vectors encoding the HIV‐1 accessory proteins Vpr, Nef, Vif, and Vpu and introduced them individually into activated primary CD4^+^ T cells (Figure 2B). Analysis at 3 days post‐transduction revealed that Vpr was uniquely sufficient to induce robust expression of *CD19, MS4A1, CD22*, and *PAX5*, whereas Nef, Vif, and Vpu had little or no effect (Figure 2C). Among these genes, *PAX5* exhibited the strongest response, increasing by more than 20‐fold compared with control cells (Figure 2C). To determine whether Vpr was also required for induction of this transcriptional program in the context of HIV‐1 infection, we generated a Vpr‐deficient HIV‐1 construct (HIV‐1‐ΔVpr) (Figure 2D). Whereas wild‐type HIV‐1 infection induced substantial upregulation of *CD19, MS4A1, CD22*, and *PAX5*, deletion of Vpr largely abolished this effect (Figure 2E). Thus, both gain‐of‐function and loss‐of‐function approaches consistently identified Vpr as the principal viral determinant responsible for induction of B cell‐associated genes in primary CD4^+^ T cells. Taken together, these findings demonstrate that Vpr is both sufficient and required for the activation of a B cell‐associated transcriptional program in primary CD4^+^ T cells and establish Vpr as a key driver of the B cell‐like phenotype observed following HIV‐1 infection.

### 2.3 Transient Induction of B Cell‐associated Genes by HIV‐1 Vpr

**FIGURE 3:**
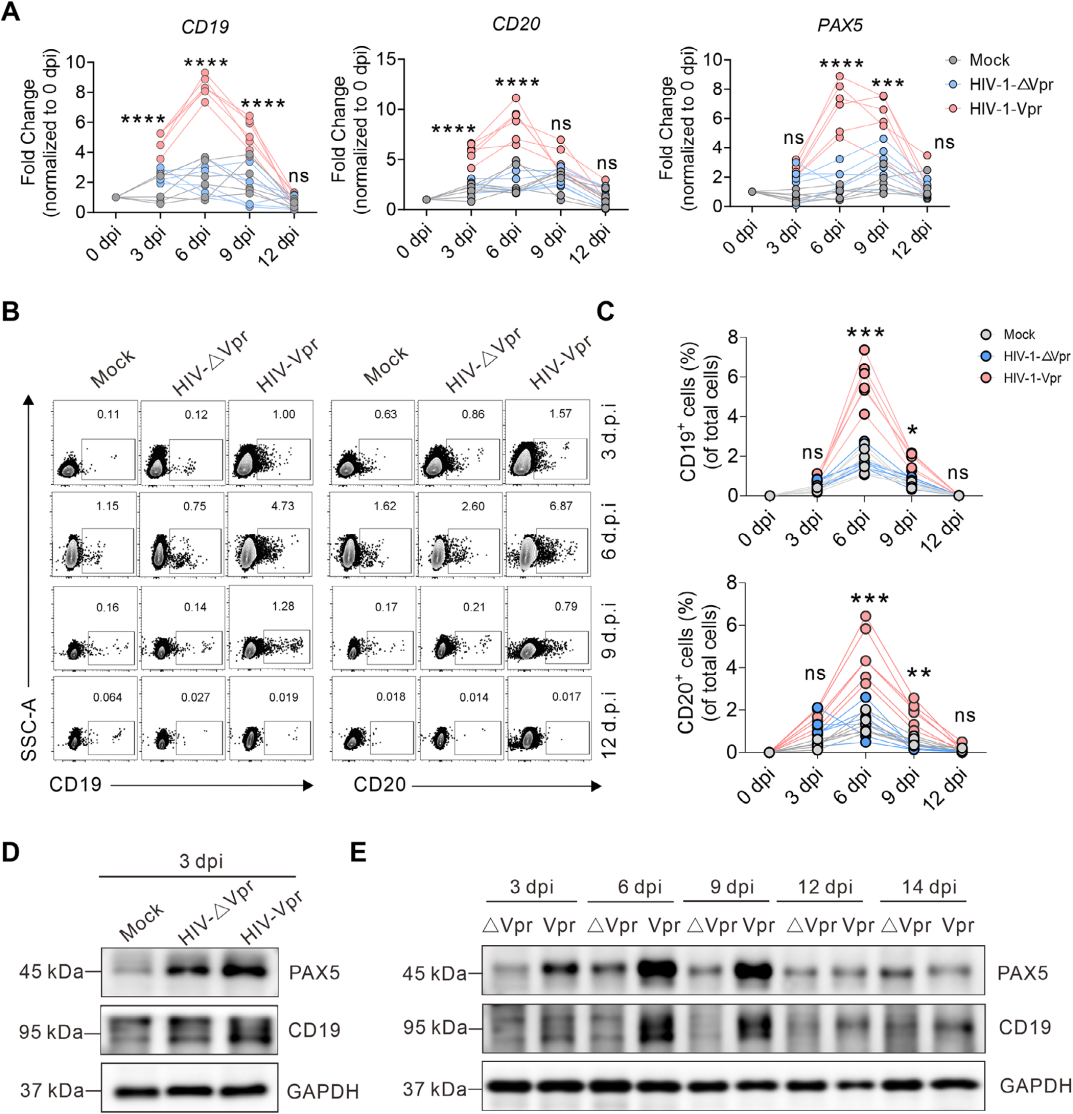
HIV‐1 Vpr induces a transient B cell‐like gene‐expression program in primary CD4^+^ T cells. (A) Time‐course analysis of *CD19, MS4A1*, and *PAX5* mRNA expression in primary CD4^+^ T cells infected with HIV‐1‐Vpr or HIV‐1‐ΔVpr (*n* = 5 donors). (B and C) Representative flow cytometry plots showing the frequency (B) and quantification (C) of CD19 and CD20 expression on Days 0, 3, 6, 9, and 12 after HIV‐1‐∆Vpr or HIV‐1‐Vpr infection and uninfected primary CD4^+^ T cells (n = 5 donors). (D) Representative immunoblot analysis of PAX5 and CD19 protein expression in primary CD4^+^ T cells 3 days after infection with HIV‐1‐Vpr or HIV‐1‐ΔVpr. (E) Time‐course immunoblot analysis of PAX5 and CD19 protein expression following HIV‐1 infection in the presence or absence of Vpr. Data are presented as mean ± s.d. In panels (A), *p*‐values were determined using an unpaired two‐tailed Student's *t* test. In panels (C) and (E), *p*‐values were determined by two‐way ANOVA with multiple‐comparison correction. ns, not significant; **p < 0.01; ***p < 0.001; ****p < 0.0001.

To determine whether Vpr‐induced expression of B cell‐associated genes was sustained over time, we monitored the expression of representative B‐cell markers following HIV‐1 infection. Quantitative PCR analysis revealed that the expression of *CD19, CD20*, and *PAX5* increased rapidly after infection, reaching maximal levels between Days 3 and 9 post‐infection before gradually returning toward baseline by Day 12 (Figure 3A). We next assessed protein expression by flow cytometry. Consistent with the expected delay between transcriptional activation and protein accumulation, no significant increase in CD19 or CD20 protein expression was observed at Day 3 post‐infection. In contrast, both markers were markedly upregulated by Day 6, followed by a progressive decline at later time points (Figure 3B,C). Similar kinetics were observed by immunoblotting, which demonstrated transient increases in total PAX5 and CD19 protein levels during Days 3–9 post‐infection, with expression returning toward baseline thereafter (Figure 3D,E). Together, these findings demonstrate that Vpr induces a transient B cell‐associated transcriptional and protein‐expression program rather than a stable lineage conversion. The temporal dynamics suggest that Vpr‐mediated remodeling is most prominent during the early stages of infection and progressively diminishes thereafter.

### 2.4 Vpr Influences Bystander B‐cell Phenotypes in a co‐culture System

The presence of Vpr outside cells has been confirmed in the serum of people living with HIV‐1 [44, 45], and anti‐Vpr antibodies have also been detected in infected individuals [46]. Multiple studies indicate that extracellular Vpr can induce cell death, suggesting its potential role in bystander cell depletion [47, 48, 49]. To explore whether Vpr‐mediated remodeling of infected CD4^+^ T cells might influence neighboring B cells, we established an autologous CD4^+^ T‐cell/B‐cell co‐culture system (Figure S2A). Activated primary CD4^+^ T cells were infected with HIV‐1‐Vpr or HIV‐1‐ΔVpr, washed extensively, and subsequently co‐cultured with autologous B cells isolated from the same donor. At Day 6 post‐co‐culture, the proportion of CD19^+^ cells was more than fivefold higher in the HIV‐1‐Vpr condition than in the HIV‐1‐ΔVpr control (Figure S2B,C). Furthermore, Ki‐67 analysis revealed a reduced frequency of proliferating CD4^+^ T cells but an increased frequency of Ki‐67^+^ CD19^+^ B cells in the presence of Vpr (Figure S2D,E), suggesting that Vpr‐associated signals may differentially influence T‐cell and B‐cell activation states. To further assess potential effects on B‐cell homeostasis, we examined expression of surface IgD and IgM. Vpr exposure reduced the proportion of IgD^+^ IgM^+^ B cells (Figure S2F,G), indicating that Vpr‐mediated signals may alter B‐cell phenotypes and potentially affect normal B‐cell differentiation or functional states. Collectively, these results suggest that, in addition to driving a transient B cell‐like transcriptional program in infected CD4^+^ T cells, Vpr may also influence neighboring B cells within the local cellular environment.

### 2.5 Vpr Drives a Conserved B Cell‐associated Transcriptional Program in both Infected and Bystander CD4^+^ T Cells

**FIGURE 4:**
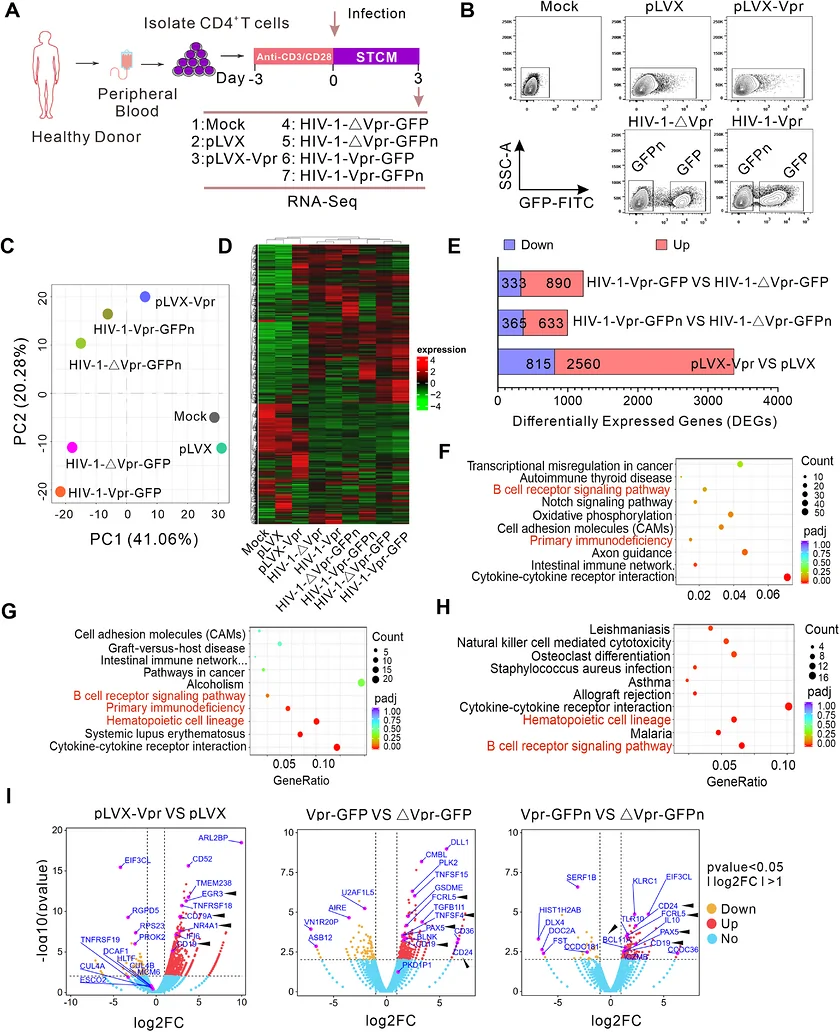
Vpr remodels the transcriptional landscape of primary CD4^+^ T cells in both infected and bystander populations. (A) Experimental design for bulk RNA‐seq. Activated primary CD4^+^ T cells from three HIV‐1‐naïve donors were infected with HIV‐1‐Vpr‐GFP or HIV‐1‐ΔVpr‐GFP viruses, or transduced with pLVX‐Vpr or empty‐vector control (pLVX). (B) Fluorescence‐activated cell sorting (FACS) strategy. Productively infected GFP^+^ cells (HIV‐1‐Vpr‐GFP and HIV‐1‐ΔVpr‐GFP) and GFP^−^ bystander cells (HIV‐1‐Vpr‐GFPn and HIV‐1‐ΔVpr‐GFPn) were isolated on day 3 post‐infection prior to RNA‐seq analysis. (C) Principal component analysis (PCA) of transcriptomic profiles from all experimental groups. (D) Heatmap showing differentially expressed genes (DEGs) identified across the indicated comparisons. Rows represent genes and columns represent samples. Colors indicate normalized expression levels (FPKM). (E) Statistical comparison of DEGs between sample groups. (F‐H) Top 10 enriched KEGG pathways among Vpr‐regulated DEGs in pLVX‐Vpr versus pLVX (F), HIV‐1‐Vpr‐GFP versus HIV‐1‐ΔVpr‐GFP (G), and HIV‐1‐Vpr‐GFPn versus HIV‐1‐ΔVpr‐GFPn (H). Pathways related to B‐cell receptor signaling, hematopoietic cell lineage, and cytokine signaling are prominently enriched. (I) Volcano plots highlighting Vpr‐regulated genes in the three comparison groups. Representative genes associated with B‐cell differentiation and lymphocyte development are indicated.

To define the transcriptional programs regulated by Vpr, we performed bulk RNA sequencing of activated primary CD4^+^ T cells from healthy donors under three complementary experimental conditions: (i) HIV‐1 infection with either wild‐type Vpr or Vpr‐deficient virus, (ii) fluorescence‐activated cell sorting (FACS)–purified productively infected and bystander populations from infected cultures, and (iii) lentiviral Vpr overexpression (Figure 4A,B). Because Vpr is packaged within virions and delivered to target cells during viral entry, its biological effects may extend beyond productively infected cells. To distinguish direct effects associated with productive infection from those occurring in bystander populations, GFP^+^ (HIV‐1‐∆Vpr‐GFP or HIV‐1‐Vpr‐GFP) and GFP^−^ (HIV‐1‐∆Vpr‐GFPn or HIV‐1‐Vpr‐GFPn) cells were isolated separately from HIV‐1‐Vpr and HIV‐1‐ΔVpr cultures prior to RNA sequencing (Figure 4A,B). Principal component analysis revealed clear transcriptional separation among the experimental groups. Notably, the transcriptional profile of pLVX‐Vpr‐infected cells clustered more closely with HIV‐1‐Vpr‐GFPn cells than with productively infected HIV‐1‐Vpr‐GFP cells (Figure 4C), suggesting that virion‐delivered Vpr can induce substantial transcriptional remodeling even in the absence of productive infection. Differential expression analysis demonstrated widespread Vpr‐dependent transcriptional changes across all experimental systems (Figure 4D,E). Importantly, Vpr induced both gene repression and gene activation, with gene induction representing a prominent component of the response. Consistent with the bystander activity of Vpr, comparison of HIV‐1‐Vpr‐GFPn and HIV‐1‐ΔVpr‐GFPn cells identified 998 differentially expressed genes, indicating that exposure to Vpr profoundly alters transcriptional programs even in cells lacking detectable productive infection. Pathway enrichment analyses revealed remarkable consistency among the three experimental systems. Vpr‐induced genes were significantly enriched in pathways associated with B‐cell receptor signaling, hematopoietic cell lineage specification, cytokine signaling, NF‐κB signaling, and PI3K‐Akt signaling (Figure 4F–I, Figure S3, Table S1). Notably, several canonical B‐cell‐associated genes, including *CD19, CD79A, PAX5, FCRL5*, and *CD24*, were among the most strongly induced transcripts (Figure 4I). Together, these findings demonstrate that Vpr drives extensive transcriptional remodeling in primary CD4^+^ T cells and consistently activates gene programs associated with lymphocyte differentiation and B‐cell identity.

**FIGURE 5:**
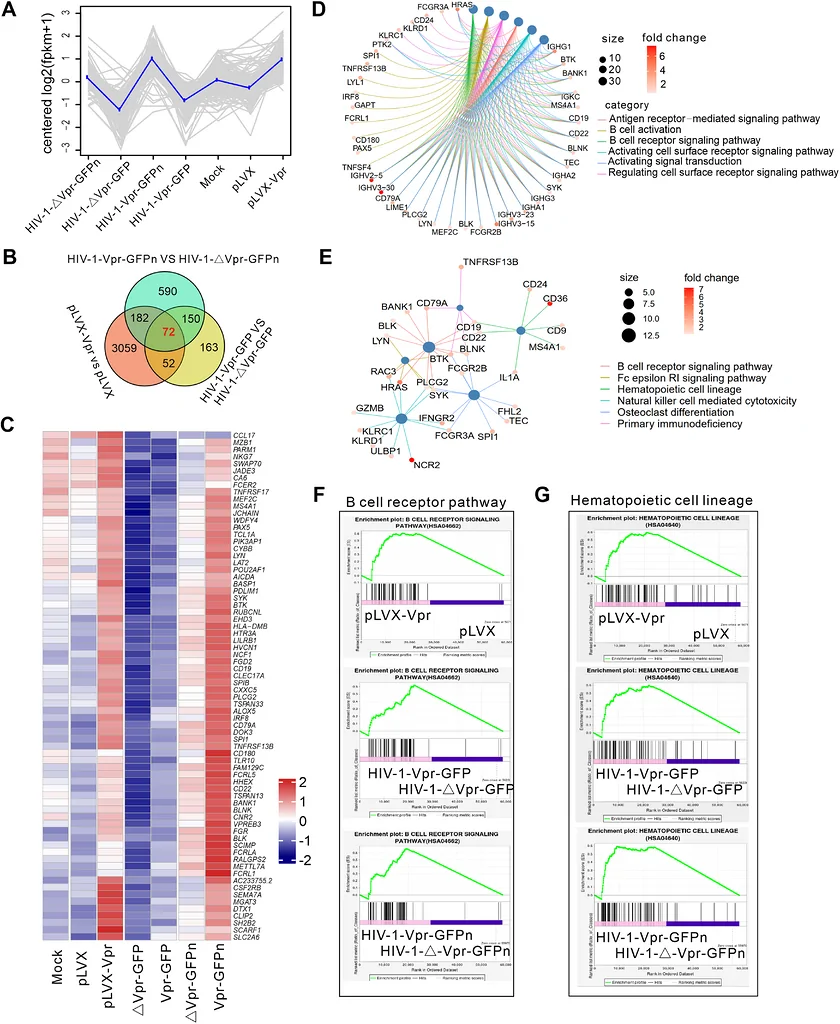
Vpr induces a shared transcriptional program enriched for B‐cell receptor signaling and lymphocyte differentiation. (A) Expression‐pattern clustering of Vpr‐upregulated DEGs identified across the indicated RNA‐seq comparisons. Gray lines represent individual genes and blue lines indicate the average expression trajectory within each cluster. Gene expression levels are plotted on the *y*‐axis, centered at log2(fpkm+1), with groups displayed on the *x*‐axis. (B) Venn diagram showing overlap of Vpr‐upregulated DEGs among pLVX‐Vpr versus pLVX, HIV‐1‐Vpr‐GFP versus HIV‐1‐ΔVpr‐GFP, and HIV‐1‐Vpr‐GFPn versus HIV‐1‐ΔVpr‐GFPn comparisons. (C) Heatmap displaying expression of the 72 shared Vpr‐upregulated genes across all experimental groups. (D) Gene‐concept network analysis of the top six enriched Gene Ontology (GO) biological processes associated with the 72 shared Vpr‐upregulated genes. (E) Gene‐concept network analysis of the top six enriched KEGG pathways associated with the 72 shared Vpr‐upregulated genes. (F) Gene Set Enrichment Analysis (GSEA) demonstrating enrichment of the B‐cell receptor signaling pathway among Vpr‐upregulated genes. (G) GSEA demonstrating enrichment of the hematopoietic cell‐lineage program among Vpr‐upregulated genes.

To identify the core transcriptional program consistently regulated by Vpr, we compared Vpr‐responsive genes across the lentiviral overexpression, productively infected, and bystander‐cell datasets. This analysis identified 72 genes that were reproducibly upregulated under all three conditions (Figure 5A,B). Interestingly, induction of these genes was strongest in pLVX‐Vpr cells and HIV‐1‐Vpr‐GFPn cells, whereas expression was comparatively attenuated in productively infected HIV‐1‐Vpr‐GFP cells (Figure 5C). These findings suggest that Vpr‐mediated transcriptional remodeling is not restricted to productively infected cells and may be partially masked by the broader transcriptional changes associated with late stages of HIV‐1 infection. Gene‐concept network analysis revealed that the shared Vpr‐responsive genes were enriched in pathways associated with B‐cell receptor signaling, antigen receptor‐mediated signaling, lymphocyte activation, and hematopoietic differentiation (Figure 5D,E). Consistent with these findings, gene set enrichment analysis confirmed significant enrichment of both the B‐cell receptor signaling pathway and hematopoietic cell lineage signatures across Vpr‐exposed populations (Figure 5F,G). Collectively, these analyses identify a conserved Vpr‐responsive transcriptional module characterized by activation of B‐cell‐associated differentiation pathways and provide transcriptome‐wide support for the emergence of a B cell‐like program in primary CD4^+^ T cells.

### 2.6 Vpr Remodels Chromatin Accessibility at Lymphocyte Differentiation Loci

**FIGURE 6:**
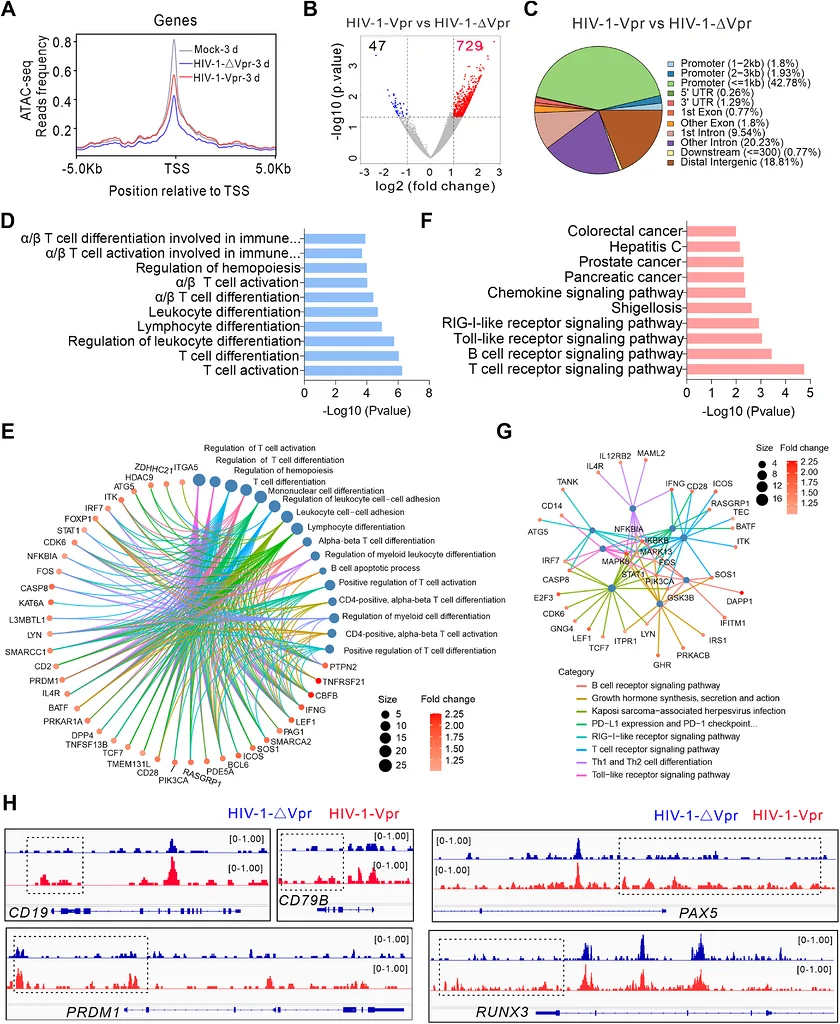
Vpr promotes chromatin accessibility at genes involved in lymphocyte differentiation and B‐cell development. (A) ATAC‐seq signal density surrounding transcription start sites (TSSs) in Mock, HIV‐1‐ΔVpr, and HIV‐1‐Vpr groups. (B) Volcano plot showing differentially accessible chromatin regions in HIV‐1‐Vpr versus HIV‐1‐ΔVpr infected primary CD4^+^ T cells. (C) Genomic annotation of differential ATAC‐seq peaks identified in the HIV‐1‐Vpr versus HIV‐1‐ΔVpr comparison. (D) Top 10 enriched GO biological processes associated with genes linked to significantly increased chromatin accessibility in the HIV‐1‐Vpr group. (E) Gene‐concept network of the top 16 GO terms related to genes associated with significantly upregulated ATAC‐seq peaks in the HIV‐1‐Vpr vs. HIV‐1‐∆Vpr comparison. (F) Top 10 enriched KEGG pathways associated with genes linked to significantly increased chromatin accessibility in the HIV‐1‐Vpr group. (G) Gene‐concept network of the top eight KEGG pathways related to genes associated with significantly upregulated ATAC‐seq peaks in the HIV‐1‐Vpr vs. HIV‐1‐∆Vpr comparison. (H) Integrative Genomics Viewer (IGV) tracks showing representative examples of Vpr‐induced chromatin opening at loci involved in B‐cell differentiation and development, including *CD19, CD79B, PAX5, PRDM1*, and *EBF1*. Dashed boxes indicate regions exhibiting increased chromatin accessibility in HIV‐1‐Vpr relative to HIV‐1‐ΔVpr infected cells.

The chromatin state plays a pivotal role in various cellular processes, including gene expression, DNA replication, and DNA repair [50]. Regulatory sequences, such as promoters, enhancers, insulators, and locus control regions, are typically associated with accessible chromatin [51]. These elements, in conjunction with cell type‐specific transcription factors, orchestrate transcriptional processes critical for determining cell fate and development [52, 53, 54]. To determine whether the transcriptional remodeling induced by Vpr was accompanied by epigenetic changes, we performed ATAC‐seq on primary CD4^+^ T cells infected with HIV‐1‐Vpr, HIV‐1‐ΔVpr, or mock controls. ATAC‐seq libraries exhibited the expected nucleosomal periodicity and strong enrichment around transcription start sites (TSSs), confirming high‐quality chromatin accessibility profiles (Figure 6A, Figure S4A). Although HIV‐1 infection globally reduced chromatin accessibility compared with mock‐treated cells, the presence of Vpr markedly increased accessibility around TSS regions relative to HIV‐1‐ΔVpr infection (Figure 6A). Consistent with this observation, differential accessibility analysis identified substantially more Vpr‐induced open chromatin regions in the HIV‐1‐Vpr versus HIV‐1‐ΔVpr comparison than in either infection‐versus‐mock comparison (Figure 6B, Figure S4B). Notably, these Vpr‐responsive regions were preferentially enriched within promoter‐proximal sequences (Figure 6C, Figure S4C), suggesting that Vpr promotes transcriptionally permissive chromatin states during HIV‐1 infection.

To explore the functional significance of Vpr‐responsive chromatin regions, we analyzed the associated differentially accessible genes (DAGs). GO and KEGG analyses consistently revealed enrichment for pathways associated with lymphocyte differentiation, lymphocyte activation, T‐cell receptor signaling, and B‐cell receptor signaling (Figure 6D–G, Figure S4D–I). Among the 729 DAGs that exhibited increased accessibility in HIV‐1‐Vpr relative to HIV‐1‐ΔVpr infection, many encoded established regulators of lymphocyte fate determination, including *FOXP1, PRDM1, BATF, TCF7, BCL6*, and *CBFB* (Tables S2 and S3). These findings indicate that Vpr selectively enhances accessibility at genomic loci involved in immune‐cell differentiation rather than inducing indiscriminate chromatin opening.

Given the prominent B cell‐like transcriptional signature identified by RNA‐seq, we next examined chromatin accessibility at key B‐cell lineage regulators. IGV visualization demonstrated increased accessibility at multiple canonical B‐cell‐associated loci, including *CD19, CD79B, PAX5, EBF1*, and *PRDM1*, in the presence of Vpr (Figure 6H, Figure S5J). Particularly notable was PRDM1 (Blimp‐1), a master regulator of plasma‐cell differentiation [55], as well as PAX5 and EBF1, two transcription factors central to B‐cell lineage specification [56, 57]. Increased accessibility was also observed at BATF, CBFB, RUNX3, IRF4, and JUND, genes known to participate in lymphocyte differentiation and immune‐cell activation [58, 59, 60, 61, 62, 63, 64, 65, 66] (Figure S5J). Together, these data demonstrate that Vpr‐driven transcriptional remodeling is accompanied by coordinated epigenetic reprogramming and establish chromatin accessibility changes as a mechanistic basis for the emergence of the B cell‐associated gene‐expression program in HIV‐1‐infected CD4^+^ T cells.

## 3 Discussion

HIV‐1 is unable to directly infect B cells because they lack the CD4 receptor required for viral entry. Nevertheless, abnormalities in B‐cell homeostasis and humoral immunity are among the most consistent immunological features of HIV‐1 infection. Recent studies have further reported the presence of CD20‐expressing HIV‐infected cells in vivo, suggesting a previously unappreciated connection between HIV‐1 infection and B‐cell‐associated phenotypes [67, 68, 69]. Interestingly, Wu et al. observed that a small population of cells annotated as B cells and monocytes emerged following HIV‐1 infection of purified primary CD4^+^ T cells, and that a subset of these cells contained HIV‐derived reads [70]. Although these populations were interpreted as potential contaminants, our findings raise the possibility that at least part of this phenomenon may reflect HIV‐induced transcriptional remodeling of infected CD4^+^ T cells. Consistent with these observations, our single‐cell multi‐omics analyses identified a distinct population of HIV‐1 RNA^+^ cells localized within B‐cell clusters and exhibiting expression of canonical B‐cell‐associated genes. We further demonstrate that this phenotype is driven by the HIV‐1 accessory protein Vpr, which induces expression of *CD19, MS4A1, CD22, PAX5*, and additional B‐cell‐associated genes in primary CD4^+^ T cells.

Vpr has long been recognized as a multifunctional accessory protein capable of manipulating numerous host‐cell processes, including cell‐cycle regulation, DNA‐damage responses, apoptosis, and degradation of cellular proteins [36, 37, 38, 39, 40, 41, 42, 43]. Although several transcriptomic studies have documented widespread Vpr‐dependent gene‐expression changes in primary CD4^+^ T cells [40, 41, 42], the biological significance of these transcriptional programs remains incompletely understood. Rather than inducing isolated marker genes, our transcriptomic analyses revealed coordinated activation of a broader B‐cell‐associated gene network. Canonical B‐cell markers such as CD19 and CD20 were induced alongside lineage‐associated regulators including PAX5, PRDM1, BCL11A, and EBF1. Pathway‐level analyses consistently identified enrichment of B‐cell receptor signaling, hematopoietic lineage specification, lymphocyte differentiation, and immune‐activation pathways. Importantly, these findings were independently validated in both Vpr‐overexpression and HIV‐1 infection systems and were further supported by re‐analysis of an independent published transcriptomic dataset. Together, these observations suggest that Vpr activates a coordinated developmental program associated with B‐cell identity rather than merely inducing expression of individual B‐cell markers.

Importantly, the transcriptional remodeling induced by Vpr was accompanied by extensive epigenetic changes. Chromatin accessibility profiling demonstrated that Vpr increased accessibility at loci encoding key regulators of lymphocyte differentiation, including PAX5, EBF1, PRDM1, BATF, RUNX1, and RUNX3. The concordance between transcriptional activation and chromatin opening suggests that the observed phenotype is supported by coordinated epigenetic remodeling rather than stochastic fluctuations in gene expression. These findings are consistent with our previous work demonstrating Vpr‐induced alterations in DNA methylation patterns of genes involved in lymphocyte differentiation and development [71]. Taken together, the present study supports a model in which Vpr reshapes both the transcriptional and epigenetic landscapes of primary CD4^+^ T cells, thereby transiently unlocking developmental programs that are normally inaccessible to mature T cells.

An important observation from our RNA‐seq analyses is that Vpr‐associated transcriptional changes were detected not only in productively infected cells but also in bystander populations. This finding is consistent with the known biology of Vpr, which is incorporated into virions and delivered into target cells during viral entry independently of productive infection. Previous studies have shown that virion‐associated Vpr can reprogram transcriptional pathways in resting or bystander T cells, thereby influencing cellular phenotypes beyond directly infected cells [42]. In agreement with these observations, we identified extensive Vpr‐dependent transcriptional differences in bystander populations and found that many Vpr‐responsive genes were induced more strongly in bystander cells and Vpr‐overexpression systems than in productively infected cells. One possible explanation is that Vpr‐mediated transcriptional programs are most readily observed when Vpr acts in relative isolation. In contrast, productively infected cells are simultaneously exposed to extensive viral gene expression, host‐transcriptional remodeling, cell‐cycle perturbation, and other virus‐induced regulatory mechanisms that may attenuate or obscure specific Vpr‐dependent effects. Although this interpretation remains speculative, it provides a plausible explanation for the differential transcriptional responses observed between infected and bystander populations and highlights the broader impact of Vpr on the immune environment beyond infected cells alone.

Emerging evidence have revealed a previously underappreciated degree of lineage plasticity in mature T cells [72, 73, 74, 75]. In our recent work, HIV‐1 infection was shown to drive the emergence of HLA class II–restricted CD8^+^ T cells from mature CD4^+^ T cells through extensive transcriptional [76], demonstrating that HIV‐1 can reshape lineage‐associated programs within the T‐cell compartment. In the present study, we extend this concept by showing that HIV‐1 Vpr induces the expression of canonical B‐cell genes, including *CD19, MS4A1, CD22, PAX5, EBF1*, and *PRDM1*, and promotes chromatin accessibility at multiple B‐lineage regulatory loci. Interestingly, the extent of reprogramming observed here appears fundamentally different from that observed in the CD4‐to‐CD8 transition. CD4^+^ and CD8^+^ T cells originate from closely related thymic developmental pathways and share much of their underlying transcriptional circuitry. In contrast, B cells and T cells diverge much earlier during lymphoid development and are maintained by distinct lineage‐specifying transcription factor networks. Consistent with this developmental distance, the Vpr‐induced B cell‐like state was incomplete and transient. These observations suggest that HIV‐1 may be considerably more capable of driving plasticity within the T‐cell lineage than across major lymphoid lineages.

The biological significance of the Vpr‐induced B cell‐like program is likely not the generation of bona fide B cells, but rather the transient rewiring of host‐cell identity during HIV‐1 infection. Although Vpr induced the expression of canonical B‐cell genes and increased chromatin accessibility at key B‐lineage regulatory loci, these changes were incomplete and reversible, indicating activation of a partial B‐cell developmental program rather than full lineage conversion. Nevertheless, such lineage mimicry may have important consequences. HIV‐1 infection is characterized by profound disruption of B‐cell homeostasis, including hypergammaglobulinemia, expansion of atypical memory B cells, impaired vaccine responses, and defective antibody maturation [12, 13]. Because B cells are not major targets of HIV‐1 infection, these abnormalities are generally attributed to indirect mechanisms. Our findings suggest an additional possibility: HIV‐1 may perturb humoral immunity by inducing B‐cell‐associated transcriptional programs in infected CD4^+^ T cells. Consistent with this notion, Vpr altered the behavior of neighboring B cells in our co‐culture system, increasing the proportion of Ki‐67^+^ B cells while reducing the frequency of IgD^+^ IgM^+^ cells. Furthermore, the emergence of CD19^+^/CD20^+^ HIV‐1 RNA^+^ cells may have implications for HIV‐1 reservoir biology, as CD20‐expressing infected‐cell populations have been reported in both experimental models and clinical studies [77, 78]. Together, these observations support a model in which Vpr‐mediated cellular reprogramming contributes to immune dysregulation and phenotypic heterogeneity of HIV‐1‐infected cells. More broadly, our findings add to growing evidence that HIV‐1 actively manipulates host‐cell identity programs and suggest that lineage plasticity may represent a previously underappreciated component of HIV‐1 pathogenesis.

Several limitations of this study should be acknowledged. First, all experiments were performed using primary cells from HIV‐1‐naïve healthy donors infected ex vivo. Because the Vpr‐induced B cell‐like program is transient and most prominent during the early phase of infection, direct validation in clinical samples would likely require access to hyperacute HIV‐1 infection cohorts before widespread viral dissemination and initiation of antiretroviral therapy. Such specimens remain difficult to obtain, limiting our ability to determine whether similar cellular states arise in vivo. Second, although our data consistently demonstrate Vpr‐dependent induction of B‐cell‐associated genes, chromatin accessibility, and transcriptional programs, these findings do not establish complete lineage conversion. Rather, our results support the existence of a reversible B cell‐like transcriptional and epigenetic state in HIV‐1 infected CD4^+^ T cells. Definitive demonstration of lineage conversion would require genetic lineage tracing, long‐term fate mapping, and comprehensive functional analyses of B‐cell identity and function, which were beyond the scope of the present study. Finally, while independent transcriptomic datasets and multiple orthogonal experimental approaches supported our observations, the molecular mechanisms by which Vpr activates B‐lineage regulatory networks remain incompletely understood. Future studies combining acute‐infection cohorts, mechanistic perturbation experiments, and single‐cell multi‐omics technologies will be required to determine whether similar cellular states occur in vivo and to define their contribution to HIV‐1 pathogenesis, immune dysregulation, and reservoir persistence.

## 4 Materials and Methods

### 4.1 Human Participants

The isolation of primary CD4^+^ T cells from healthy adult peripheral blood mononuclear cells was conducted with approval from the Institutional Ethics Committee of the Third Affiliated Hospital of Sun Yat‐sen University (Guangzhou, Guangdong, China; approval number [2022]02‐199‐01). All participants provided written informed consent for both participation in the study and the publication of their experimental data.

### 4.2 Cell Lines

HEK293T cells were maintained in Dulbecco's Modified Eagle Medium (DMEM, Gibco) supplemented with 10% heat‐inactivated fetal bovine serum (FBS, Sigma‐Aldrich) and 1 U/mL penicillin‐streptomycin (Gibco). The cells were cultured at 37°C in a humidified atmosphere containing 5% CO_2_ for pseudovirus production. The identity of the HEK293T cell line was authenticated by short tandem repeat (STR) profiling, and the corresponding STR authentication report was provided in supplemental material.

### 4.3 Virus Stock Production

Pseudotyped viral stocks were generated in HEK293T cells through co‐transfection with 2.2 µg of the pCXCR4 HIV‐1 envelope expression vector, 4.4 µg of the packaging vector pC‐help, and 4.4 µg of the HIV‐1 pseudotyped vector using a polyethyleneimine (Invitrogen) transfection system, following the manufacturer's protocol. Lentivirus for overexpression was similarly produced in HEK293T cells by co‐transfecting 2.2 µg of the VSV‐G glycoprotein expression vector, 4.4 µg of the packaging vector pC‐help, and 4.4 µg of the lentiviral vector. Supernatants were collected 48 h post‐transfection, centrifuged at 500 × g for 10 min at room temperature, and filtered through a 0.45 µM pore membrane to eliminate cellular debris. Viral particles were concentrated via centrifugation with 25% (v/v) 50% polyethylene glycol 6000 and 10% (v/v) 4 M NaCl. The concentrated virions were resuspended in complete medium and stored at ‐80°C.

### 4.4 HIV‐1 Infection and Cell Culture

Primary CD4^+^ T cells were isolated from HIV‐1 naïve individuals. Fresh PBMCs were labeled using the BD IMag Human CD4^+^ T Lymphocyte Enrichment Set‐DM (#557939, BD Biosciences) for negative selection. Isolated CD4^+^ T cells were activated with anti‐CD3 (#300302, BioLegend) and anti‐CD28 antibodies (#302902, BioLegend) for 3 days in super T cell medium (STCM). Activated CD4^+^ T cells were then infected with pseudotyped viral stocks at a multiplicity of infection (MOI) of 0.05–0.1. All infections were performed by centrifuging the target cells with the virus at 1200 × g for 2 h. Post‐infection, the cells were cultured in STCM for 3 days, after which the medium was replaced with cytokine‐free medium (CFM) for extended culture. CFM: cytokine‐free medium, CFM consisted of RPMI 1640 supplemented with 10% FBS and 1% penicillin‐streptomycin and contained no exogenous cytokines or growth factors; STCM: super T cell medium, CFM with IL‐2 (PeproTech, Cat# 200‐02‐50UG) and TCGF (T cell growth factor, it was produced as previously reported [79].

### 4.5 Plasmid Construction

The coding sequences (CDS) of the HIV‐1 accessory proteins Vpr, Vif, Nef, and Vpu were amplified by PCR from the HIV‐1 NL4‐3 proviral backbone using gene‐specific primers harboring pre‐designed restriction enzyme sites. The amplified CDS fragments and the pLVX lentiviral expression vector were subjected to double restriction enzyme digestion with the corresponding endonucleases, followed by ligation with T4 DNA ligase (NEB# M0202) to generate the overexpression constructs pLVX‐Vpr, pLVX‐Vif, pLVX‐Nef, and pLVX‐Vpu. Individual colonies were picked and screened, and all constructs were verified by Sanger sequencing prior to use. The Vpr‐deficient HIV‐1 construct (HIV‐1‐ΔVpr) was generated in the same proviral background.

### 4.6 Flow Cytometry

Surface staining was performed using fluorochrome‐conjugated antibodies, including anti‐CD4‐APC‐Cy7 (BioLegend, Cat#317418), anti‐CD19‐APC (BioLegend, Cat# 302212), anti‐CD20‐PE (BioLegend, Cat# 302306), anti‐IgM‐PE‐Cy7 (Elabscience, Cat# E‐AB‐F1172D), anti‐IgD‐FITC (Elabscience, Cat# E‐AB‐F1171C), were used, and a viability dye was included to exclude dead cells. The flow cytometry data were acquired using a BD LSR Fortessa cell analyzer and sorted using a FACS Aria II (BD). Data were analyzed using FlowJo software (version 10.4.0).

### 4.7 Immunoblotting

Lysates from 1 × 10^6^ cells were prepared for protein expression analysis via immunoblotting. The cells were resuspended in 1× RIPA buffer supplemented with protease inhibitors. Protein concentrations were quantified using the BCA protein assay kit (BCA, #P0009D, Beyotime). Total cellular proteins were resolved by SDS‐PAGE, transferred onto membranes, and analyzed by immunoblotting. Detection and visualization were performed with Image Lab software on a ChemiDoc XRS+ system (Bio‐Rad). Primary antibodies against PAX5 (Abcam, Cat# ab109443) and CD19 (Abcam, Cat# ab237772), GAPDH (Abcam, Cat# ab181602) together with the corresponding loading‐control antibody, were used for immunoblotting.

### 4.8 DNA, RNA Extraction, and qPCR

Total DNA was extracted using the Tissue DNA Kit (OMEGA), and total RNA was extracted from total infected cultures with TRIzol reagent (Invitrogen) and chloroform, followed by precipitation with isopropyl alcohol. Reverse transcription of RNA to cDNA was performed according to the protocol for SuperScript IV Control Reactions (Thermo Fisher Scientific). Relative quantitative PCR was conducted with SYBR qPCR Master Mix (Vazyme) on the QuantStudio 3 system (Thermo Fisher Scientific) using a standard two‐step protocol. Primer sequences are provided in Table S4. The 2^−ΔΔCT^ method was employed to analyze the relative expression levels of mRNAs, with GAPDH serving as the reference gene. Absolute quantitative PCR was performed using the TaqMan Gene Expression Assay (Life Technologies).

### 4.9 RNA‐Seq Experiments and Analysis

Total RNA was extracted from the indicated cell populations using TRIzol Reagent (Invitrogen) according to the manufacturer's instructions. For HIV‐1 infection experiments, GFP^+^ productively infected cells and GFP^−^ bystander cells were purified by fluorescence‐activated cell sorting (FACS) prior to RNA isolation. RNA‐seq libraries were generated using the TruSeq Stranded mRNA Library Preparation Kit (Illumina) following the manufacturer's protocol and sequenced on an Illumina NextSeq 500 platform. Raw sequencing reads were aligned to the human reference genome (GRCh38) using STAR (v2.5.1b), and transcript abundance was quantified with RSEM (v1.2.22). Gene expression levels were calculated as transcripts per million (TPM) and normalized across samples using the upper‐quartile normalization method. Differentially expressed genes (DEGs) were identified using DESeq2. Genes with an adjusted *p* value < 0.05 and an absolute log2 fold change > 1 were considered significantly differentially expressed unless otherwise specified. Principal component analysis (PCA), hierarchical clustering, Gene Ontology (GO), Kyoto Encyclopedia of Genes and Genomes (KEGG), and Gene Set Enrichment Analysis (GSEA) were performed using standard R packages.

### 4.10 ATAC‐Seq Experiments and Analysis

Infected cells were collected, and 50,000 cells were pretreated with DNase at 37°C for 30 min to remove extracellular DNA and degrade DNA from dead cells. The cells were then centrifuged at 500 × g for 5 min, and the resulting pellet was resuspended in 50 µL of cold lysis buffer. The cell lysis reaction was incubated on ice for 3 min. Following lysis, 1 mL of ATAC‐seq resuspension buffer was added, and the nuclei were centrifuged at 500 × g for 10 min. The nuclei were resuspended in 50 µL of transposition mix containing 10 µL of 5 × TD buffer, 5 µL of Tn5 transposase, and 35 µL of water. The transposition reaction was performed at 37°C for 30 min in a thermomixer with shaking at 1000 rpm. The reactions were subsequently purified using Zymo DNA Clean and Concentrator 5 columns, and the DNA was eluted in 25 µL of 2 × HiFi PCR mix with 1 µL each of Nextera i5 and i7 primers. PCR amplification was performed using the following thermal cycling conditions: initial denaturation at 72°C for 5 min, 98°C for 1 min, followed by 14 cycles of 98°C for 15 s, 60°C for 30 s, and 72°C for 1 min. Size selection of the amplified DNA was conducted using EpiTM DNA Clean Beads (Epibiotek, cat. no. R1809). First, 35 µL of beads was added, and the supernatant was transferred to a new tube, followed by the addition of 10 µL of fresh beads to isolate fragments in the range of 250–350 bp. Library quantification was performed using a Bioptic Qsep100 Analyzer (Bioptic Inc.), and paired‐end sequencing with a read length of 150 bp was conducted. The sequencing data were analyzed by Guangzhou Epibiotek Co., Ltd. Quality control, alignment, and enrichment analyses were carried out using the ENCODE ATAC‐seq pipeline. Paired‐end reads of 150 bp were mapped to the human reference genome (hg38), and differential accessible regions were identified using the DiffBind R package.

### 4.11 Single‐cell RNA Sequencing, TCR Sequencing, and BCR Sequencing

Freshly isolated PBMCs from healthy donors were activated with anti‐CD3/CD28 antibodies for 3 days and subsequently infected with HIV‐1‐ΔE‐GFP. At the indicated time points, viable cells were sorted using a BD FACSAria II cell sorter and processed for single‐cell library preparation using the Chromium Single Cell 5′ platform (10x Genomics). Libraries were sequenced on an Illumina NovaSeq platform. Raw scRNA‐seq data were processed using Cell Ranger (v5.0.0, 10x Genomics). To identify HIV‐1–infected cells, a customized reference genome was generated by combining the human reference genome (GRCh38) with the HIV‐1 NL4‐3 genome using the Cell Ranger “mkref” pipeline. Sequencing reads were aligned to the customized reference genome, and feature‐barcode matrices were generated using the Cell Ranger “multi” pipeline. Cells containing HIV‐1 transcripts above the predefined threshold (HIV RNA counts ≥ 24) were classified as HIV‐1 RNA^+^ cells, as previously reported [76]. Downstream analyses were performed using Seurat (v4.0.6). Low‐quality cells were excluded based on standard quality‐control metrics. Data were normalized, scaled, and subjected to dimensionality reduction, unsupervised clustering, and UMAP visualization. Cell clusters were annotated according to established lineage‐specific marker genes. Differentially expressed genes (DEGs) were identified using the Wilcoxon rank‐sum test with thresholds of log2 fold change > 0.25 and false discovery rate (FDR) < 0.05. RNA velocity analysis was performed using scVelo (v0.2.2). For single‐cell TCR and BCR sequencing, raw V(D)J sequencing data were processed using Cell Ranger (v5.0.0, 10x Genomics) and aligned to the human GRCh38 V(D)J reference database. TCR and BCR clonotypes were assembled using the Cell Ranger V(D)J pipeline, enabling identification of paired receptor chains and complementary determining region 3 (CDR3) sequences. Clonotype analyses and visualization were performed using Loupe V(D)J Browser (v2.0.1, 10x Genomics).

### 4.12 Quantification and Statistical Analysis

Statistical analyses were performed using GraphPad Prism (v9.2.0; GraphPad Software). The number of biological replicates (*n*) and the statistical tests used for each experiment are indicated in the corresponding figure legends. Data are presented as mean ± standard deviation (s.d.). Comparisons between two groups were performed using unpaired two‐tailed Student's *t*‐tests. Comparisons involving multiple groups or multiple time points were analyzed using two‐way ANOVA, or mixed‐effects models, as appropriate. p‐values < 0.05 were considered statistically significant. Statistical significance is indicated as follows: ns, not significant; *p < 0.05; **p < 0.01; ***p < 0.001; ****p < 0.0001.

## Author Contributions

J.C. and K.D. designed this study. J.C. and K.D. supervised this study. P.W., J.C., Z.M., W.W., H.W., and S.D. performed the experiments. J.C. and J.L. performed the data analysis. J.C. and J.L. wrote the manuscript with inputs from other authors. All authors have read and approved the final manuscript.

## Funding

This work was supported by the Prevention and Control of Emerging and Major Infectious Diseases‐National Science and Technology Major Project (grant no.: 2025ZD01904600), the National Natural Science Foundation of China (grant no.: 82302514), and the Shenzhen Medical Research Fund (grant no.: B2402023).

## Ethics Statement

The use of human peripheral blood from healthy adult donors was approved by the Institutional Ethics Committee of the Third Affiliated Hospital of Sun Yat‐sen University (Guangzhou, Guangdong, China; approval number [2022]02‐199‐01), and all participants provided written informed consent for participation and for publication of their data. This study did not involve animal experiments; animal‐ethics approval is therefore not applicable.

## Conflicts of Interest

The authors declare no conflicts of interest.

## Supporting information




**Supporting file 1**: mco271018‐sup‐0001‐SuppMat.pdf

**Supporting file 2**: mco271018‐sup‐0002‐TableS1.xlsx

**Supporting file 3**: mco271018‐sup‐0003‐TableS2.xlsx

**Supporting file 4**: mco271018‐sup‐0004‐TableS3.xlsx

**Supporting file 5**: mco271018‐sup‐0005‐TableS5.xlsx

## Data Availability

The original data files of the single cell, RNA‐seq and ATAC‐seq are available in Genome Sequence Archive at https://ngdc.cncb.ac.cn/gsa‐human/, reference number HRA005936. The data deposited and made public are compliant with the regulations of the Ministry of Science and Technology of China. Other data are available from the corresponding author upon reasonable request.
