# Supplementary material for: HIV‐1 Vpr Drives a B Cell‐Like Phenotype in Primary CD4+ T Cells
Source: MedComm (2020). 2026 Sep 27;7(10):e71018. doi: 10.1002/mco2.71018 (PMC13616185; doi:10.1002/mco2.71018)
Supplement: Supplementary file 1 [file MCO2-7-e71018-s003.pdf]

HIV-1 Vpr drives a B cell-like phenotype in primary CD4⁺ T cells

Peipei Wang[1], Jieying Li[2], Wei Wang[3,4], Zhuoyue Meng[3,4], Huixin Wu[3,4], Shumin Du[5], Kai Deng[3,4,6]*, Jinfeng Cai[3,4]*

[1]Department of Infectious Diseases, The Third Affiliated Hospital, Sun Yat-sen University, Guangzhou, China

[2]Basic Medical Experimental Teaching Center, Zhongshan School of Medicine, Sun Yat-sen University, Guangzhou, China

[3]Institute of Human Virology, Key Laboratory of Tropical Disease Control of Ministry of Education, Zhongshan School of Medicine, Sun Yat-sen University, Guangzhou, China

[4]Department of Immunology and Microbiology, Zhongshan School of Medicine, Sun Yat-sen University, Guangzhou, China

[5]Core Facility of Medical Science, Sun Yat-sen University, Guangzhou, China

[6]Advanced Medical Technology Center, The First Affiliated Hospital, Zhongshan School of Medicine, Sun Yat-sen University, Guangzhou, China

*Correspondence: caijf9@mail.sysu.edu.cn; dengkai6@mail.sysu.edu.cn.

Supplementary Materials

The PDF file includes:

Figures S1 to S4

Tables S1 to S5

STR authentication report

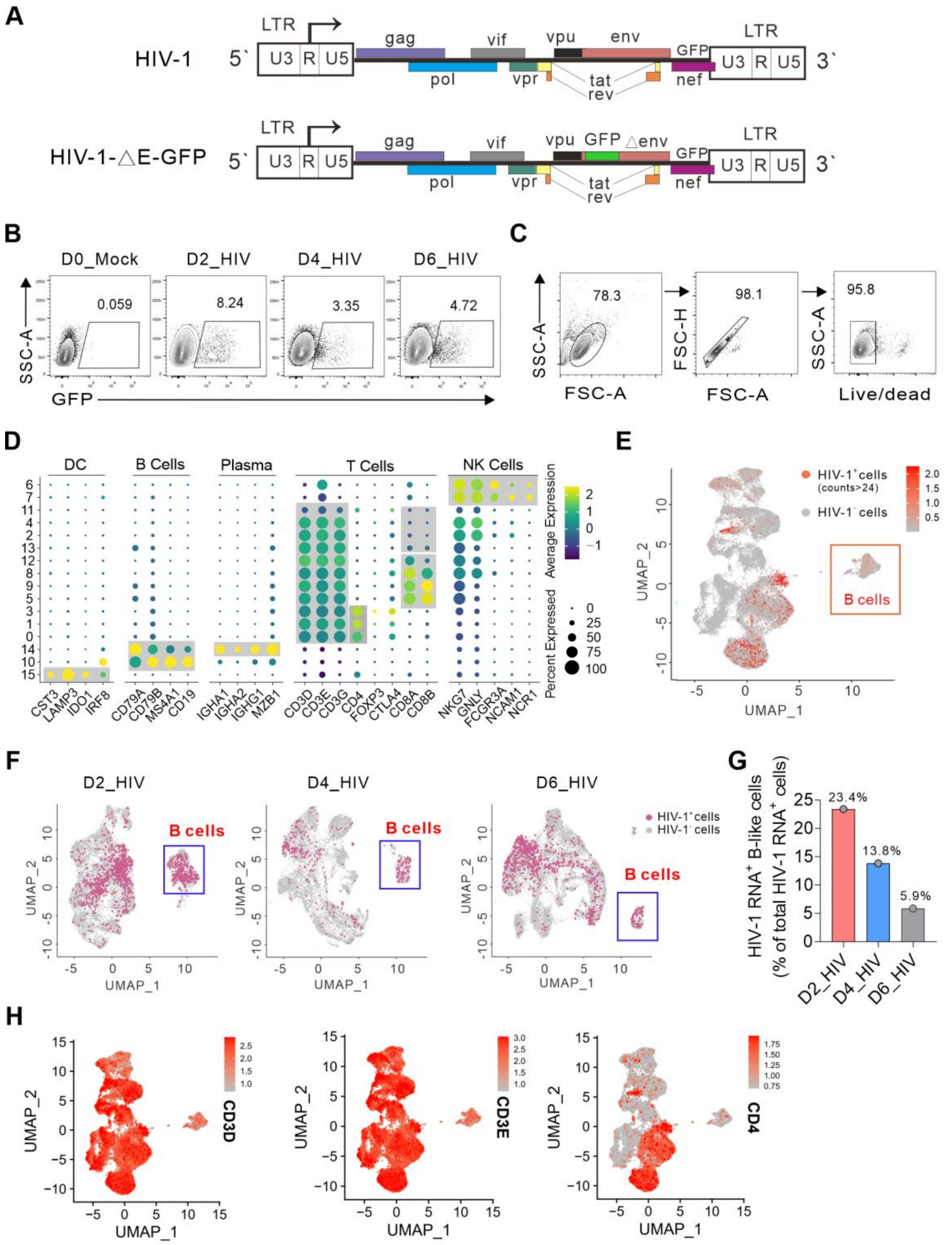

Figure S1. Single-cell transcriptomic analysis identified a population of HIV-1 RNA⁺ B-like cells during HIV-1 infection. (A) Diagram of the derivation of HIV-1-△E-GFP from the original NL4-3 strain of HIV-1. (B) Flow cytometry plots showing the frequency of infected cells on days 0, 2, 4, and 6 after HIV-1-△E-GFP infection. (C) Gating strategy used for live-cell sorting before single-cell sequencing. (D) Dot plot heatmap depicting the expression levels and cellular detection rates of selected cluster marker genes across all samples. (E) UMAP plot highlighting HIV-1 RNA⁺ cells

identified by detection of HIV-1 transcripts within the single-cell datasets. The intensity of red indicates the abundance of HIV-1 transcripts. (F) UMAP plots highlighting HIV-1 RNA$^+$ cells identified by detection of HIV-1 transcripts in D2_HIV, D4_HIV, and D6_HIV samples. Red dots represent HIV-1 RNA$^+$ cells. (G) Proportion of HIV-1 RNA$^+$ B-like cells relative to the total population of HIV-1-infected cells at D2_HIV, D4_HIV, and D6_HIV. (H) UMAP plots showing single-cell transcript levels of *CD3D*, *CD3E*, and *CD4*. Transcript levels are color-coded: gray indicates no expression, while red denotes expression.

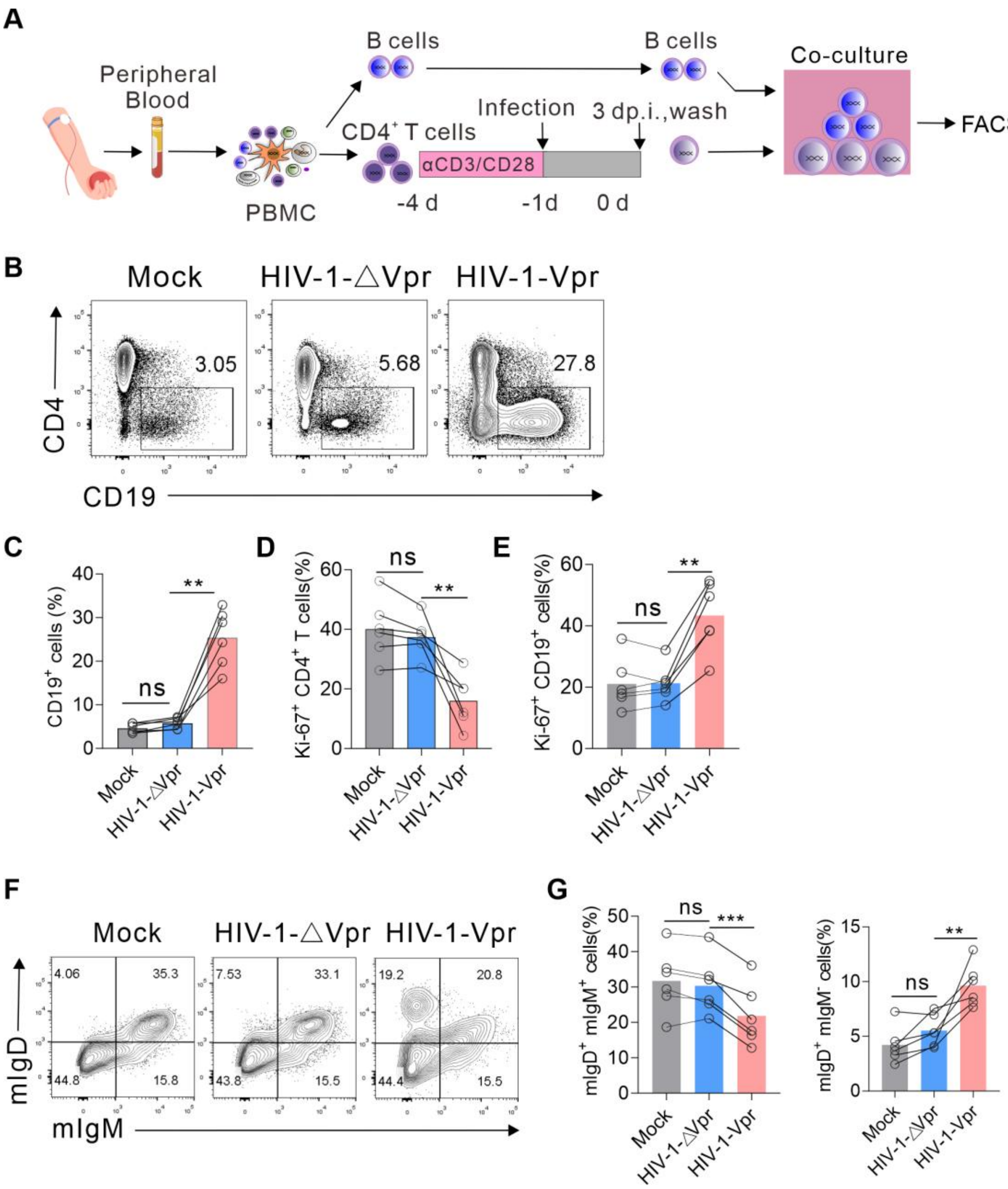

Figure S2. Vpr modulates B-cell proliferation and surface immunoglobulin expression. (A) Overview of the workflow and experimental design for co-culture. Activated $CD4^+$ T cells were infected with HIV-1-Vpr or HIV-1-ΔVpr, washed extensively, and subsequently co-cultured with autologous B cells. (B, C) Representative flow cytometry plots showing the frequency (B) and quantification (C) of CD19 expression on day 6 after co-culture (n=6 donors). (D, E) Quantification of $Ki67^+$ $CD4^+$ T cells (D) and $Ki67^+$ B cells (E) on days 6 after co-culture (n=6 donors). (F, G) Representative flow cytometry plots showing the frequency (F) and quantification (G) of mIgD and mIgM expression on B cells after 6 days of co-culture (n=6 donors). Data are presented as mean ± s.d. Statistical significance was determined using two-way ANOVA (or mixed-effects model). ns, not significant; $^{**}p < 0.01$; $^{***}p < 0.001$.

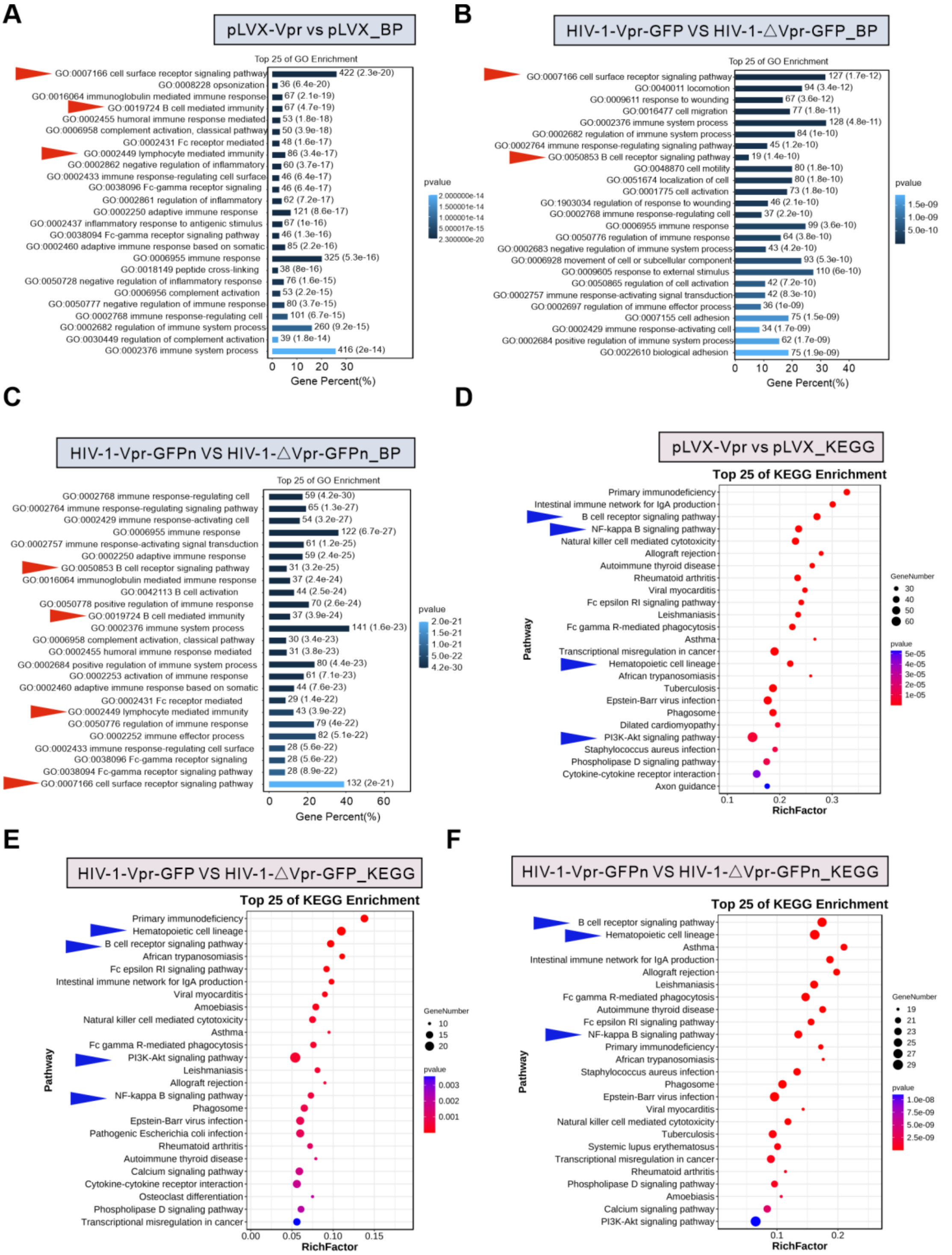

Figure S3. Characterization of Vpr-induced upregulated DEGs. (A-C) Biological process analysis of Vpr-induced upregulated differentially expressed genes (DEGs) in the pLVX-Vpr vs. pLVX group (A), HIV-1-Vpr-GFP vs. HIV-1-ΔVpr-GFP group (B), and HIV-1-Vpr-GFPn vs. HIV-1-ΔVpr-GFPn group (C). (D-F) Top 25 KEGG pathway enrichment analysis of Vpr-induced upregulated DEGs in the pLVX-Vpr vs. pLVX group (D), HIV-1-Vpr-GFP vs. HIV-1-ΔVpr-GFP group (E), and HIV-1-Vpr-GFPn vs. HIV-1-ΔVpr-GFPn group (F).

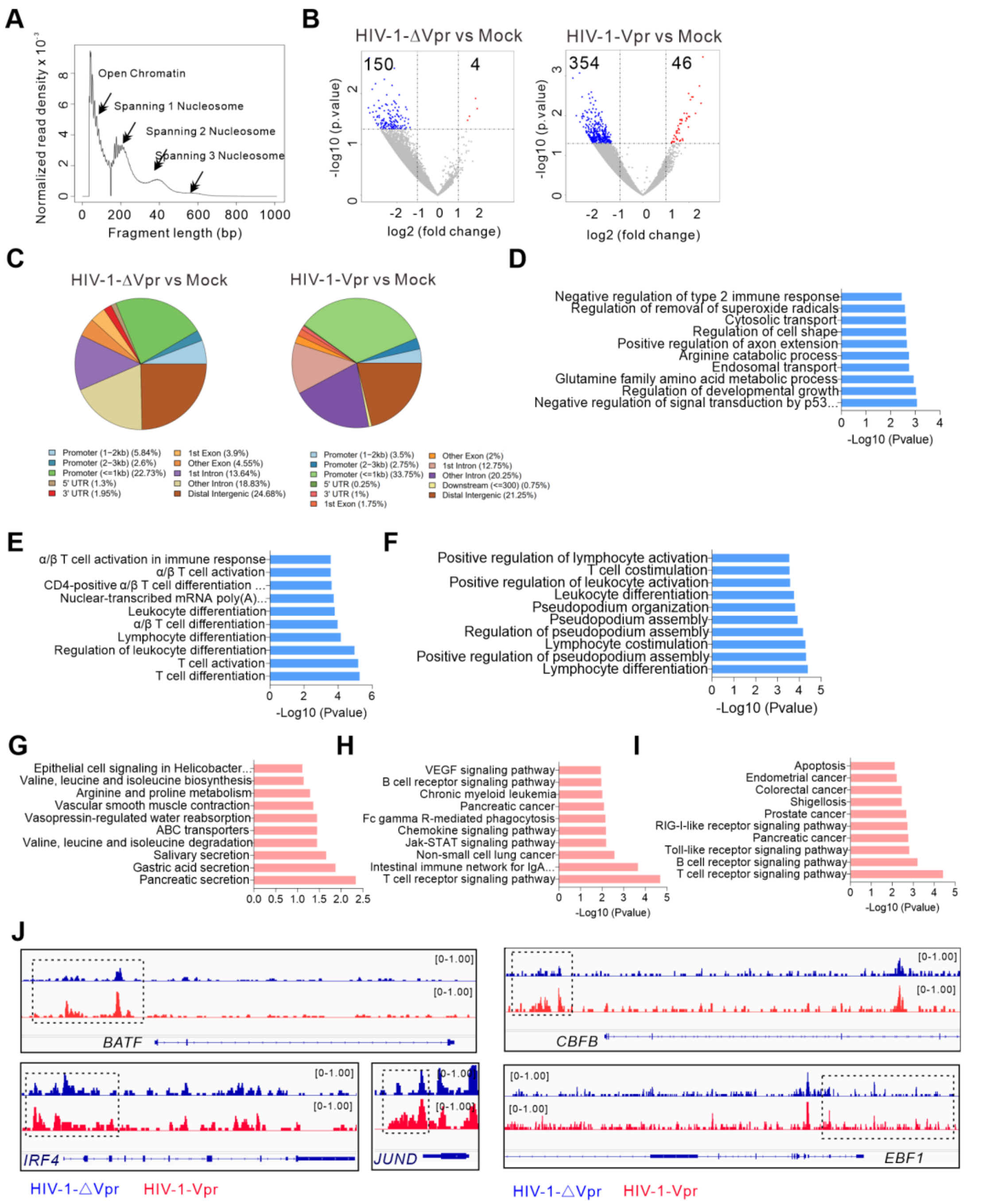

Figure S4. Vpr promotes widespread chromatin remodeling in primary CD4^{+} T cells. (A) Fragment sizes within a representative ATAC-seq library. Smaller fragments correspond to sequence reads from open chromatin, with the peak around ~150 bp representing sequence reads that span a single nucleosome, while larger peaks indicate progressively more compact chromatin structures. (B) Volcano plot depicting ATAC-seq peaks identified in HIV-1-ΔVpr versus Mock (left panel) and HIV-1-Vpr versus Mock (right panel). (C) Pie charts illustrating the distribution of genomic features among the differential peaks in HIV-1-ΔVpr versus Mock (left panel), and HIV-1-Vpr versus Mock (right panel). (D-F) Top ten biological processes associated with genes linked to differential peaks in the HIV-1-ΔVpr versus Mock (D), HIV-1-Vpr versus

Mock (E), and HIV-1-Vpr versus HIV-1-ΔVpr (F) groups. (G-I) Top ten KEGG pathways associated with genes linked to differential peaks in the HIV-1-ΔVpr versus Mock (G), HIV-1-Vpr versus Mock (H), and HIV-1-Vpr versus HIV-1-ΔVpr (I) groups. (J) IGV snapshots showing the ATAC-seq signal at five representative loci involved in T-lymphocyte differentiation (BATF, CBFB, RUNX3, IRF4, and JUND). Dashed boxes highlight regions with increased chromatin accessibility in response to HIV-1-Vpr.

Table S1. Vpr-induced differentially expressed genes (DEGs) in the pLVX-Vpr vs. pLVX, HIV-1-Vpr-GFP vs. HIV-1-ΔVpr-GFP, and HIV-1-Vpr-GFPn vs. HIV-1-ΔVpr-GFPn (separate Excel file).

Table S2. GO analysis of Vpr-induced differentially accessible genes (DAGs) in HIV-1-Vpr vs. HIV-1-ΔVpr (separate Excel file).

Table S3. KEGG analysis of Vpr-induced differentially accessible genes (DAGs) in HIV-1-Vpr vs. HIV-1-ΔVpr (separate Excel file).

Table S4. The sequences of primers used in qPCR

Primer	Sequence
GAPDH-F	CTCTGCTCCTCCTGTTCGAC
GAPDH-R	AGTTAAAAGCAGCCCTGGTGA
CD19-F	GGCCCGAGGAACCTCTAGT
CD19-R	TAAGAAGGGTTTAAGCGGGGA
MS4A1-F	CCGGCAGAGCCAATGAAAG
MS4A1-R	CAGATGGGTGCATAGATCCCT
CD22-F	GCACCCTGAAACCCTCTACG
CD22-R	ATCAAACTTCGAGGTGTTCTTGT
PAX5-F	ACTTGCTCATCAAGGTGTCAG
PAX5-R	TCCTCCAATTACCCCAGGCTT

Table S5. Full unedited blot for Figure 3D and Figure 3E (separate Excel file).

STR authentication report
